# LncRNA LINC00662 Exerts an Oncogenic Effect on Osteosarcoma by the miR-16-5p/ITPR1 Axis

**DOI:** 10.1155/2021/8493431

**Published:** 2021-09-28

**Authors:** Miao Yu, Weihao Lu, Zhenglin Cao, Tianhang Xuan

**Affiliations:** Department of Spine Surgery, Foshan Hospital of Traditional Chinese Medicine, Foshan 528000, Guangdong, China

## Abstract

**Background:**

Osteosarcoma (OS) is one of the most malignant bone tumors and has a high metastatic rate. Increasing research has demonstrated the vital roles of long noncoding RNAs (lncRNAs) in human cancers, including OS. LncRNA LINC00662 has been revealed to act as an oncogene involved in multiple tumor progression. This study aimed to investigate the expression pattern, function, and regulatory mechanism of LINC00662 in OS.

**Methods:**

Patients who underwent OS surgery were involved in this study. Experiments including RT-qPCR, MTT, western blot, FISH, RNA pull-down, luciferase reporter, colony formation, transwell invasion and migration, and sphere formation assay were performed to investigate the regulatory role of LINC00662 in OS.

**Results:**

In the present study, our findings demonstrated the upregulation of LINC00662 expression in OS tissues and cells, and high expression of LINC00662 predicted a poor clinical prognosis of patients' iNOS. Through a series of *in vivo* assays, LINC00662 knockdown suppressed OS cell proliferation, invasion, migration, and stemness property maintenance. Further mechanistical investigations indicated that LINC00662 functioned as a competing endogenous RNA (ceRNA) for sponging microRNA-16-5p (miR-16-5p) to upregulate the expression of IP receptor type 1 (ITPR1) in OS cells. Restoration assays validated the involvement of ITPR1 in LINC00662-mediated regulation of cell functions in OS.

**Conclusion:**

LINC00662 exerts oncogenic functions in OS by targeting the miR-16-5p/ITPR1 axis.

## 1. Introduction

OS is a common malignant bone tumor with rapid metastasis, affecting a multitude of people, frequently during adolescence [1–3]. High genetic instability and extreme genome are two main factors contributing to the occurrence and development of OS [4]. Despite significant advances in clinical therapies including surgery, chemotherapy, and radiotherapy, a long-term survival rate for patients with OS remains unsatisfying [5]. Notably, the 5-year survival rate decreases from 77.3% to 33.9% when metastasis occurs in OS patients [6]. Considering that the pathogenesis of OS is quite complex [7], it is urgent to investigate the molecular mechanisms underlying OS progression for improvement of the diagnosis and treatment.

Long noncoding RNAs (lncRNAs) are a kind of molecules longer than 200 nucleotides and lack protein-coding ability [8], playing crucial roles in various biological processes via different mechanisms [9]. Accumulating evidence has revealed the regulatory roles of lncRNAs in tumor progression. For example, lncRNA ASB16-AS1 mediates the proliferation and invasion of hepatocellular carcinoma cells by regulating the miR-1827/FZD4 axis [10]. LncRNA CCAT1 promotes colorectal cancer cell proliferation *in vitro* and *in vivo* by upregulating TUSC3 [11]. Multiple lncRNAs have been discovered to participate in OS progression. For example, lncRNA NR2F1-AS1 is overexpressed in OS, and its knockdown suppresses cell proliferation by inducing cell cycle arrest and promotes cell apoptosis [12]. LncRNA SND1-IT1 sponges miR-665 to upregulate POU2F1 expression, thereby facilitating OS cell proliferation and migration [13]. LncRNA LINC00662 has also been reported to be a key regulator of biological behaviors in many cancers. LINC00662 is upregulated in hepatocellular carcinoma and contributes to cell proliferation and invasion through activating Wnt/*β*-catenin signaling pathway [14]. LINC00662 acts as a miR-34a sponge to promote the tumorigenesis of prostate cancer [15]. LINC00662 has been considered a valuable prognostic biomarker in colon cancer [16]. Since the oncogenic effect of LINC00662 has been well clarified in other cancers, we hypothesized that LINC00662 might play a role in OS. Moreover, LINC00662 has been reported to function as a competing endogenous RNA (ceRNA) by sponging miRNAs in cancers. We thus investigated whether LINC00662 could exert ceRNA functions in OS.

In this research, we aimed to investigate the expression pattern of LINC00662 in OS clinical samples and the function of LINC00662 in OS cell proliferation, migration, invasion, and stemness characteristics, as well as the potential regulatory mechanisms of LINC00662 in OS, which might provide a novel insight for OS diagnosis and treatment.

## 2. Materials and Methods

### 2.1. Clinical Tissues

OS tissues and adjacent normal tissues from 51 patients who underwent surgical resection were collected from Foshan Hospital of Traditional Chinese Medicine (Guangdong, China) from January 2014 to December 2017. The enrolled patients were within the age of 26–65, with an average age of 45.87 ± 11.02, and they were at different TNM stages (I–VI). All patients were clinically and pathologically diagnosed. None of patients had received any antitumor therapy including radiotherapy and chemotherapy before the operation. The collected tissues were immediately frozen with liquid nitrogen and stored at −80°C until use. All patients signed written informed consent. This study was approved by the Ethics Committee of Foshan Hospital of Traditional Chinese Medicine (Guangdong, China). Follow-up of the patients via phone or return visit was performed every 3 months during the first year after surgery until December 2019 with the observation of overall survival rate of these patients.

### 2.2. Cell Culture

OS cell lines (U2OS, SAOS‐2, 143B, and MG63) and a human osteoblast cell line HFOB 1.19 were all purchased from Cell Bank of Chinese Academy of Sciences (Shanghai, China). Cells were incubated in RPMI-1640 medium (Thermo Fisher Scientific, USA) supplemented with 10% fetal bovine serum (FBS: HyClone, Shanghai, China), 100 *μ*g/mL streptomycin (Gibco, USA), and 100 U/mL penicillin (Sigma-Aldrich, USA). The medium was renewed every day. Cells were maintained in a humid incubator at 37°C with 5% CO_2_.

### 2.3. Cell Transfection

Short hairpin RNA (shRNA) targeting LINC00662 (sh-LINC0062; 5′-GCUGCUGCCACUGUAAUAATT-3′) and nontargeting shRNA negative control (sh-NC) were purchased from Sangon Biotech Co., Ltd. (St. Louis, USA). MiRNA-16-5p mimics and negative control mimics (NC mimics) were synthesized at GenePharma (Shanghai, China). The pcDNA3.1 vector containing ITPR1 (pcDNA3.1/ITPR1) and the empty vector (pcDNA3.1) were obtained from Genechem (Shanghai, China). Cell transfection was performed using Lipofectamine 3000 (Thermo Fisher Scientific, Inc., USA). The transfection efficiency was examined by RT-qPCR 48 h later.

### 2.4. RNA Extraction and Quantitative Real-Time PCR (RT-qPCR)

Extraction of total RNAs from OS tissues and cells (U2OS and SAOS-2) was performed using TRIzol reagent (Invitrogen, CA, USA). After quantification with NanoDrop 2000 (Thermo Fisher Scientific), RNA samples were reverse-transcribed into cDNA using M-MLV Reverse Transcriptase (Promega, USA). SYBR Green Master Mixture (Takara, Dalian, China) on an ABI 7500 system (Applied Biosystems, USA) was used to evaluate the expression levels of LINC00662 and ITPR1. GAPDH acted as the internal control. SYBR PrimeScript miRNA RT-PCR Kit (Takara) on an ABI 7500 system was used to detect the expression of miR-16-5p. U6 acted as the internal control. Thermal cycles were as follows: 95°C for 30 s, 95°C for 5 s for 40 cycles, and 60°C for 35 s. Relative expression was detected using the 2^−ΔΔCt^ method. The primers used in this study are shown as follows:

  LINC00662: Forward, 5′-CACGCTTCTGAAACTGGTGT-3′
  Reverse, 5′-TGTACAGCCTGGTGACAGAG-3′
  ITPR1: Forward, 5′-GAAGGCATCTTTGGAGGAAGT-3′
  Reverse, 5′-ACCCTGAGGA-AGGTTCTG-3′
  MiR-16-5p: Forward, 5′-TCCACTCTAGCAGCACGTAAAT-3′
  Reverse, 5′-TCACACTAAAGCAGCACAGTAAT-3′
  U6: Forward, 5′-CTCGCTTCGGCAGCACA-3′
  Reverse, 5′-AACGCTTCACGAATTTGCGT-3′
  GAPDH: Forward, 5′-ACAACTTTGGTATCGTGGAAGG-3′
  Reverse, 5′-GCCATCACGCCACAGTTTC-3′

### 2.5. MTT Assay

U2OS or SAOS‐2 cells were seeded in 96-well plates (5 × 10^3^ cells/well) in RPMI-1640 medium containing 10% FBS at 37°C. After incubation for 0, 24, 48, and 72 h, cells were treated with 0.5 mg·mL^−1^ MTT reagent (Sigma-Aldrich, USA). Then, cells were incubated for another 4 h. Next, the formazan precipitate was dissolved with 100 *μ*L dimethylsulfoxide (DMSO; Sigma-Aldrich). The absorbance at 570 nm was detected using a spectrophotometer (Thermo Fisher Scientific, USA).

### 2.6. Colony Formation Assay

After transfection, U2OS or SAOS‐2 cells were seeded in 6-well plates (2 × 10^5^ cells/well). The medium was replaced every 3 days. Cells were cultured for 14 days at 37°C before the medium was removed. Then, cells were washed with phosphate buffer saline (PBS). Afterwards, the colonies were fixed in 10% paraformaldehyde for 10 min and stained with 0.1% crystal violet solution (Beyotime, shanghai, China) for 10 min. The number of colonies was calculated with an inverted microscope.

### 2.7. Transwell Invasion and Migration Assays

Migratory and invasive ability of OS cells were assessed using a 24-well transwell chamber (8 *μ*m; Costar, Boston, MA, USA). For invasion detection, after transfection, 2 × 10^5^ U2OS or SAOS‐2 cells in serum-free medium were added to the upper chamber precoated with Matrigel (BD Biosciences, CA, USA), while the lower chamber was filled with 600 *μ*L RPMI-1640 medium containing 10% FBS. After 36 h of transfection, the invaded cells were fixed with 70% methanol and stained with 0.1% crystal violet (Sigma-Aldrich, USA). For migration detection, the upper chamber was not precoated with Matrigel.

### 2.8. Western Blot Analysis

The lysates of U2OS or SAOS‐2 cells were obtained using an RIPA lysis buffer (Beyotime), and protein quantification was performed using a BCA protein assay kit (Pierce, Rockford, USA). Total proteins were separated by 12% SDS-PAGE gel and transferred onto polyvinylidene fluoride (PVDF) membranes (Millipore, MA, USA), which were blocked with 5% skimmed milk for 1 h at room temperature. Then, primary antibodies (all from Boster, Wuhan, China) were incubated with the membranes overnight at 4°C. Afterwards, the membranes were washed with TBST three times and incubated with horseradish peroxidase (HRP)-conjugated secondary antibodies (Cell Signaling Technology) for 2 h as per the supplier's protocols. Then, bands were visualized with enhanced chemiluminescence (PerkinElmer, China). The primary antibodies used are shown as follows: CD44 (A00052); Oct-4 (A00174-1); Nanog (A00153-3); GAPDH (A00227); ITPR1 (PB9225).

### 2.9. Sphere Formation Assay

U2OS and SAOS‐2 cells (2.5 × 10^3^ cells/well) were seeded in 6-well ultralow adhesion plates (Corning Incorporated, USA) in serum-free RPMI-1640 medium containing 10 ng/ml epidermal growth factor (Gibco, USA), 0.5 mg/ml hydrocortisonum (Gibco), 5 mg/mL insulin (Gibco), and bovine pituitary extract (Gibco). The number of spheres (diameter >100 *μ*m) was counted under a microscope (Nikon Corporation) after two weeks.

### 2.10. RNA Fluorescence In Situ Hybridization (FISH)

The LINC00662-specific RNA FISH probe was commercially designed and synthesized by RiboBio (Guangzhou, China). A FISH Kit (RiboBio) was used to determine the probe signals. In brief, U2OS, SAOS‐2, or HFOB 1.19 cells were fixed in 4% formalin for 15 min. The cells were hybridized again in hybridization solution at 37°C for 30 min after prehybridization in PBS. Next, DAPI staining (Beyotime) was performed to counterstain the cell nuclei. The images of staining were captured using a fluorescence microscope (Leica, Germany).

### 2.11. Luciferase Reporter Assay

Wild-type LINC00662 or mutant LINC00662 sequence (mutant at the miR-16-5p binding site), and wild-type or mutant-type ITPR1′-UTR sequence were inserted into the pmirGLO vector (Promega, USA) to generate LINC00662-Wt/Mut vectors and ITPR1-Wt/Mut vectors. Afterwards, U2OS and SAOS‐2 cells were cotransfected with NC mimics or miR-16-5p mimics and the above constructed vectors using Lipofectamine 3000 Reagent (Thermo Fisher Scientific). The luciferase activities were measured using luciferase reporter assay system (Promega).

### 2.12. RNA Pull-Down Assay

LINC00662 biotin probe and LINC00662 no-biotin probe, as well as miR-16-5p biotin probe and miR-16-5p no-biotin probe, were synthesized by Sangon (Shanghai, China). The cell protein extracts from U2OS or SAOS‐2 were collected for incubation with the RNA or NC probes overnight. Then, the lysate was added with streptavidin agarose beads (Life Technologies) and incubated for another 1 h at room temperature. Next, these beads were boiled in SDS buffer and purified by RNeasy Mini Kit (Qiagen, USA). The purified RNA was detected by RT-qPCR.

### 2.13. Statistical Analysis

All data are presented as the mean ± standard deviation (SD). Statistical analysis was performed with SPSS 19.0 software (SPSS, Chicago, USA). Statistical differences between two groups were analyzed using student's *t*-test and those among groups using one-way analysis of variance (ANOVA). *P* < 0.05 was considered statistically significant. All experiments were conducted more than three times.

## 3. Results

### 3.1. LINC00662 is Upregulated in OS Tissues and Cells

**Figure 1:**
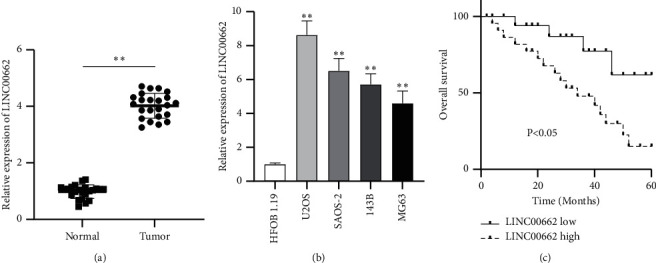
LINC00662 is upregulated in OS tissues and cells. (a) The expression of LINC00662 in 51 OS tissues and adjacent normal tissues was evaluated by RT-qPCR. (b) The expression status of LINC00662 in OS cell lines (U2OS, SAOS‐2, 143B, and MG63) and normal human line HFOB 1.19 was measured using RT-qPCR. (c) Overall survival rate of patients with OS using log‐rank test and Kaplan–Meier analysis. ^*∗∗*^*P* < 0.01.

Design of the present study is shown in Supplementary Figure 1. To determine the role of LINC00662 in OS, we first detected the expression of LINC00662 in 51 paired OS tissues and adjacent normal tissues using RT-qPCR. The results showed that LINC00662 expression was higher in OS tissues than in normal tissues (^*∗∗*^*P* < 0.01; Figure 1(a)). Subsequently, the expression of LINC00662 in OS cells was measured. As expected, LINC00662 expression was significantly upregulated in OS cell lines (U2OS, SAOS‐2, 143B, and MG63) compared with the human osteoblast cell line HFOB 1.19 (^*∗∗*^*P* < 0.01; Figure 1(b)). U2OS cells exhibited the highest level of LINC00662, followed by SAOS‐2 cells. Therefore, U2OS and SAOS‐2 cells were used for further assays. To investigate whether LINC00662 expression is correlated to the clinicopathological characters of patients, 51 patients were divided into a low-LINC00662-expression group (*n* = 25) and a high-LINC00662-expression group (*n* = 26) according to the median LINC00662 expression. As shown in Table 1, the high-LINC00662 level was significantly related to distant metastasis (*P* < 0.001), TNM stage (*P* < 0.001), and tumor size (*P*=0.007). To further analyze whether LINC00662 could act as a prognostic biomarker for OS, a clinical follow-up study was conducted to determine the overall survival of patients. Kaplan–Meier analysis indicated that the patients in the high-LINC00662-expression group had shorter overall survival compared with those in the low-LINC00662-expression group (Figure 1(c)). In univariate analysis, distant metastasis, TNM stage, tumor size, and increased LINC00662 expression were potential risk factors of shorter overall survival (*P*=0.009; *P*=0.040; *P*=0.015; *P*=0.023). After adjustment for confounding factors, the multivariate analysis indicated that metastasis and increased LINC00662 expression (*P*=0.012; *P*=0.013) were independently associated with shorter overall survival in OS patients (Table 2).

### 3.2. LINC00662 Knockdown Inhibits Malignant Phenotypes of OS Cells

**Figure 2:**
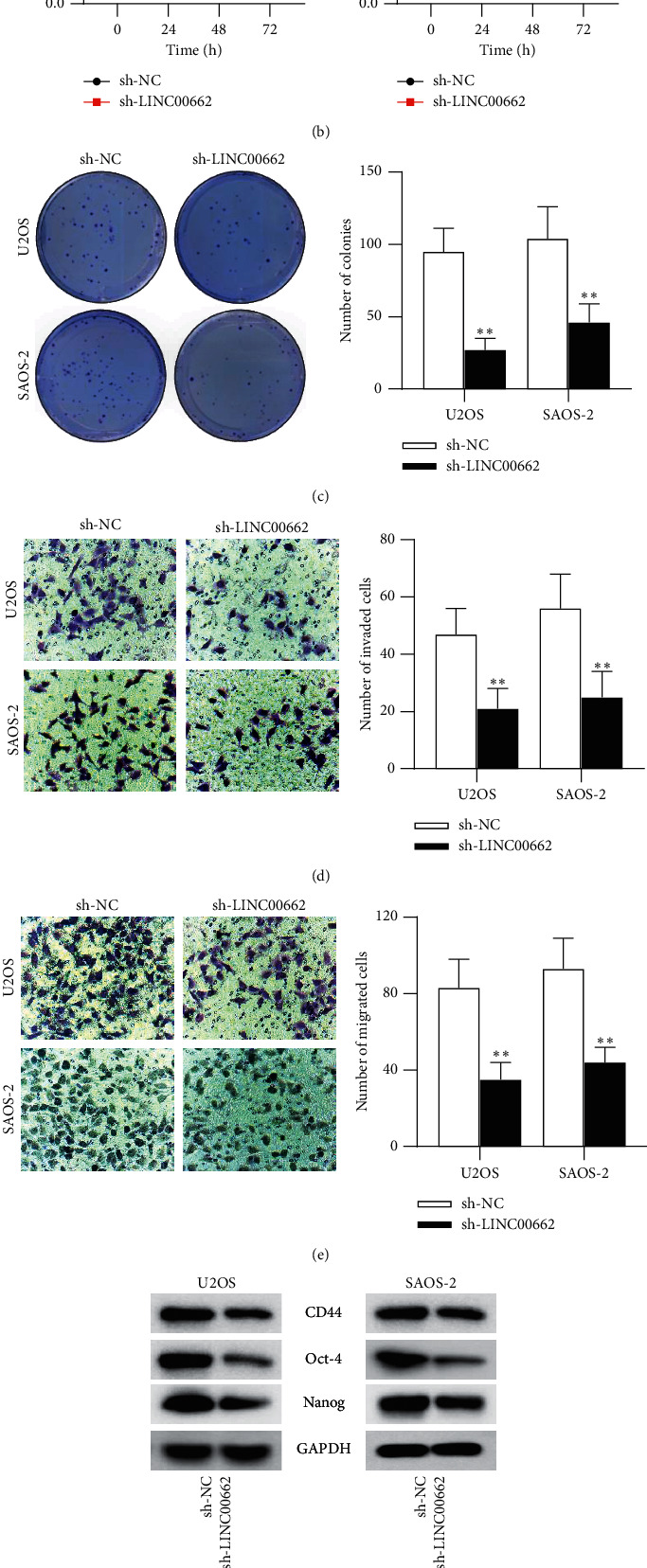
LINC00662 knockdown inhibits the malignant phenotypes of OS cells. (a) The transfection efficiency of sh-LINC00662 in U2OS and SAOS‐2 cells was measured by RT-qPCR. (b-c) Cell viability and proliferation in U2OS and SAOS‐2 cells transfected with sh-LINC00662 were examined by MTT and colony formation assays. (d-e) Transwell invasion and migration assays were performed to assess cell invasion and migration in U2OS and SAOS‐2 cells transfected with sh-LINC00662. (f-g) Western blot analysis and sphere formation assay were conducted to examine the impact of sh-LINC00662 in OS stem cell properties. ^*∗∗*^*P* < 0.01.

Since the high expression level of LINC00662 was confirmed, we then intended to explore the role of LINC00662 in OS. RT-qPCR analysis was utilized to check the transfection efficiency of sh-LINC00662 and showed that LINC00662 expression was effectively knocked down in U2OS and SAOS‐2 cells after transfection (^*∗∗*^*P* < 0.01; Figure 2(a)). The results of MTT and colony formation assays revealed that cell viability and proliferation were significantly inhibited in U2OS and SAOS‐2 cells after silencing LINC00662 (^*∗∗*^*P* < 0.01; Figures 2(b) and 2(c)). Transwell assays confirmed that inhibition of LINC00662 reduced the invasive and migratory abilities of OS cells (^*∗∗*^*P* < 0.01; Figures 2(d) and 2(e)). Additionally, the effect of LINC00662 downregulation on OS stem cell properties was investigated. Western blot analysis showed that the transfection of sh-LINC00662 decreased the levels of stemness markers (CD44, Oct-4, and Nanog) (Figure 2(f)). Furthermore, sphere formation assay demonstrated that the number of spheroids in U2OS and SAOS‐2 cells was reduced by sh-LINC00662 (^*∗∗*^*P* < 0.01; Figure 2(g)). Overall, LINC00662 inhibits tumorigenic activity in OS cells.

### 3.3. LINC00662 Acts as a Sponge for miR-16-5p

**Figure 3:**
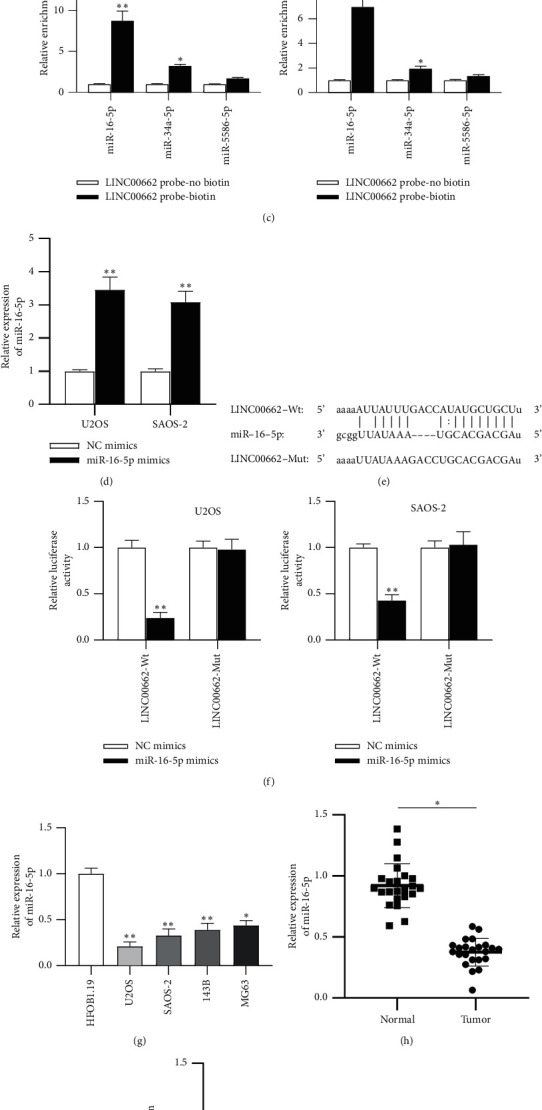
LINC00662 acts as a sponge for miR-16-5p. (a) The subcellular localization of LINCOO662 in U2OS and SAOS‐2 cells was detected using FISH. (b) The possible miRNAs which might bind to LINC00662 were predicted at the starBase website (search category: Pan-Cancer: 10 cancer types). (c) The enrichment of miRNAs in U2OS and SAOS‐2 cells was measured using RNA pull-down followed by RT-qPCR. (d) RT-qPCR was carried out to evaluate the transfection efficiency of miR-16-5p mimics in U2OS and SAOS‐2 cells. (e) The binding site between LINC00662 and miR-16-5p. (f) Luciferase activities of U2OS and SAOS‐2 cells after cotransfection with wild‐type or mutant LINC00662 vectors and miR-16-5p mimics or NC mimics. (g) The expression level of miR-16-5p in OS cell lines (U2OS, SAOS‐2, 143B, and MG63) and normal human line HFOB 1.19 was detected using RT-qPCR. (h) The expression of miR-16-5p in 51 OS tissues and adjacent normal tissues was evaluated by RT-qPCR. (i) The influence of miR-16-5p mimics on LINC00662 expression was examined by RT-qPCR in U2OS and SAOS‐2 cells. ^*∗*^*P* < 0.05; ^*∗∗*^*P* < 0.01.

To investigate how LINC00662 exerts its function, FISH assay was conducted to determine the subcellular localization on LINC00662 in OS cells. The results showed that the majority of LINC00662 was expressed in the cytoplasm of U2OS and SAOS-2 cells, and LINC00662 staining in HFOB 1.19 cells acted as a negative control (Figure 3(a)). Numerous reports indicate that cytoplasmic lncRNA could interact with miRNA to release mRNA by serving as a ceRNA [17]. We thus examined whether LINC00662 could serve as a ceRNA in OS cells. starBase (http://starbase.sysu.edu.cn/index.php) was examined to identify the potential miRNAs. We found three miRNAs (miR-16-5p, miR-34a-5p, and miR-5586-5p) containing binding site on LINC00662 sequence (search category: Pan-Cancer: 10 cancer types) (Figure 3(b)). Then, RNA pull-down assay was used to test the binding between LINC00662 and miRNAs, and the results showed that miR-16-5p was most significantly enriched in the LINC00662 probe-biotin group (^*∗*^*P* < 0.05, ^*∗∗*^*P* < 0.01; Figure 3(c)). Next, the transfection efficiency of miR-16-5p mimics in U2OS and SAOS-2 cells was confirmed by RT-qPCR (^*∗∗*^*P* < 0.01; Figure 3(d)).  Figure 3(e) shows the sequences of miR-16-5p, wild‐type LINC00662, and mutant LINC00662. It was revealed by luciferase reporter assay that miR-16-5p overexpression inhibited the luciferase activity of vectors carrying wild type rather than mutant binding site of LINC00662 in U2OS and SAOS-2 cells (^*∗∗*^*P* < 0.01; Figure 3(f)). In addition, we found that miR-16-5p was downregulated in OS cell lines (^*∗*^*P* < 0.05, ^*∗∗*^*P* < 0.01; Figure 3(g)) and tissues (^*∗*^*P* < 0.05; Figure 3(h)). Moreover, the downregulated LINC00662 expression was observed in U2OS and SAOS-2 cells after transfection with miR-16-5p mimics (^*∗*^*P* < 0.05; Figure 3(i)). The above findings indicated that LINC00662 can interact with miR-16-5p in OS cells.

### 3.4. ITPR1 is Directly Targeted by miR-16-5p

**Figure 4:**
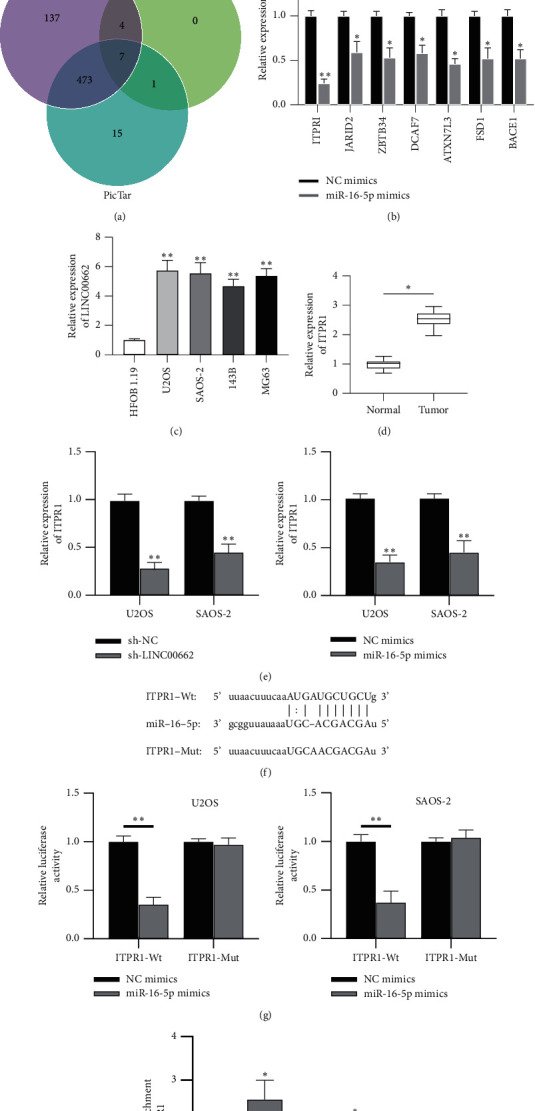
ITPR1 is directly targeted by miR-16-5p. (a) The possible mRNAs binding to miR-16-5p were predicted by PITA, RNA22, and PicTar databases. (b) The expression of candidate mRNAs in U2OS and SAOS‐2 cells transfected with miR-16-5p mimics was measured by RT-qPCR. (c-d) RT-qPCR was performed to determine the expression of ITPR1 in OS cell lines and tissues. (e) The impact of sh-LINC00662 or miR-16-5p mimics in ITPR1 expression was examined by RT-qPCR. (f) The binding site between miR-16-5p and ITPR1 obtained from starBase. (g) Luciferase reporter assay was used to test the interaction between miR-16-5p and ITPR1. (h) Interaction of miR-16-5p with ITPR1 in U2OS and SAOS‐2 cells was confirmed by RNA pull‐down assay. ^*∗*^*P* < 0.05; ^*∗∗*^*P* < 0.01.

To further explore the ceRNA pattern in OS, three online tools (PITA, RNA22, and PicTar) were searched to predict the potential targets of miR-16-5p. Venn diagram displays seven mRNAs (ITPRI, JARID2, ZBTB34, DCAF7, ATXN7L3, FSD1, and BACE1) that might bind to miR-16-5p (Figure 4(a)). RT-qPCR revealed that ITPR1 was significantly downregulated in OS cells transfected with miR-16-5p mimics compared with the other mRNAs (^*∗*^*P* < 0.05, ^*∗∗*^*P* < 0.01; Figure 4(b)). Furthermore, ITPR1 expression was revealed to be upregulated in OS cell lines and tissues (^*∗*^*P* < 0.05, ^*∗∗*^*P* < 0.01; Figures 4(c) and 4(d)). Meanwhile, ITPR1 expression at mRNA and protein levels was reduced by LINC00662 knockdown or miR-16-5p overexpression, indicating that ITPR1 is positively regulated by LINC00662 and negatively regulated by miR-16-5p (^*∗∗*^*P* < 0.01; Figure 4(e)). The binding site of miR-16-5p at the 3′‐UTR of ITPR1 is shown (Figure 4(f)). Then, luciferase reporter and RNA pull-down assays were conducted to verify their targeted relationship. As shown in Figure 4(g), the luciferase activity of ITPR1-Wt was significantly inhibited by miR-16-5p mimics, whereas that of ITPR1-Mut presented little change (^*∗*^*P* < 0.01). ITPR1 was enriched in the complex pulled down by biotinylated miR-16-5p probe (^*∗*^*P* < 0.05; Figure 4(h)), suggesting that ITPR1 is a target gene of miR-16-5p.

### 3.5. ITPR1 Reverses the Regulatory Effect of LINC00662 Knockdown in OS Cells

**Figure 5:**
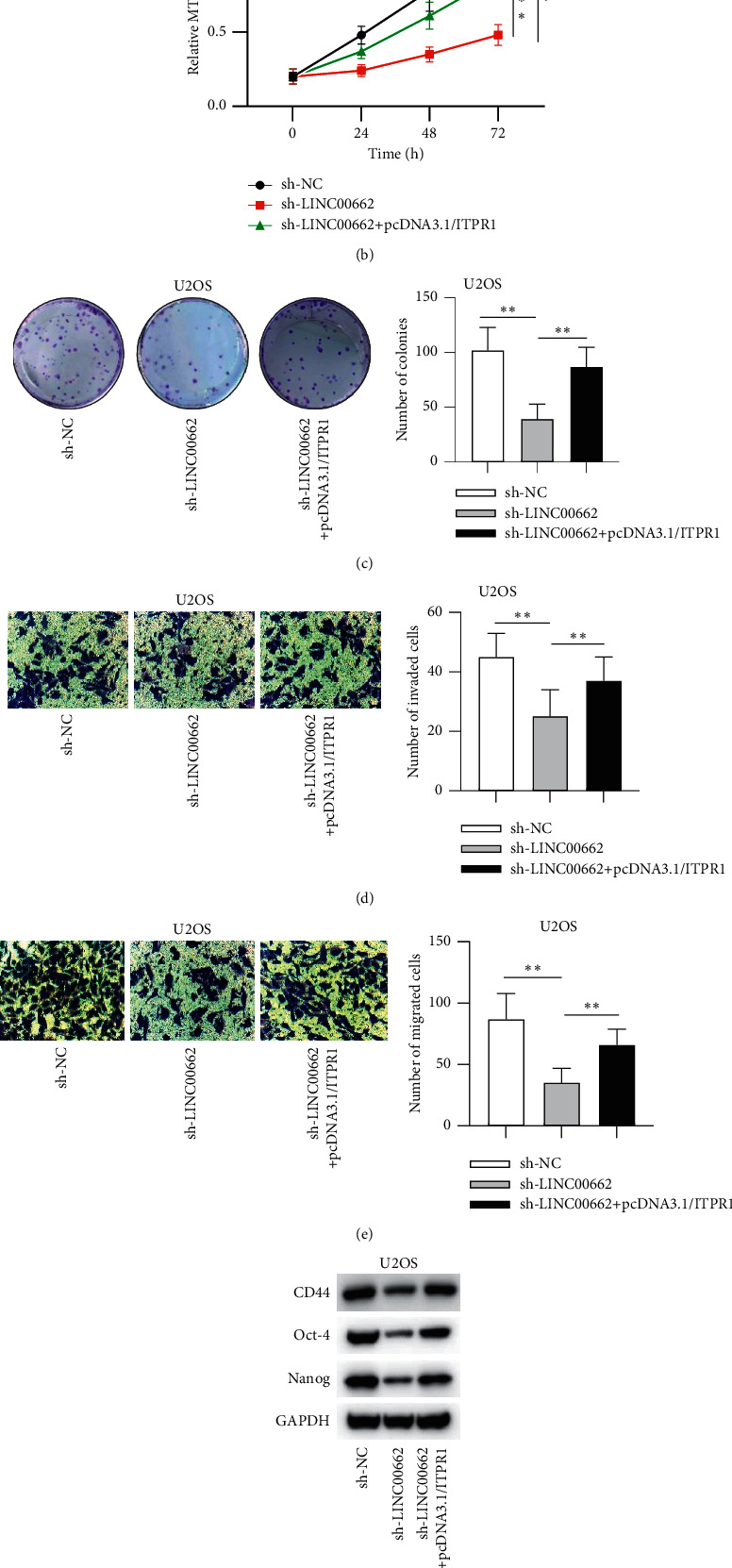
ITPR1 reverses the regulatory effect of LINC00662 knockdown in OS cells. (a) U2OS cells were transfected with sh-NC, sh-LINC00662, or sh-LINC00662 + pcDNA3.1/ITPR1, and the expression of ITPR1 in the transfected cells was measured by RT-qPCR. (b-c) Cell viability and proliferation in the transfected U2OS cells were detected using MTT and colony formation assays. (d-e) Cell invasion and migration in the transfected U2OS cells were evaluated by transwell invasion and migration assays. (f-g) Stem cell properties in the transfected U2OS cells were detected by western blot and sphere formation assays. ^*∗∗*^*P* < 0.01.

To further test whether ITPR1 expression is responsible for the function of LINC00662, the expression of ITPR1 at mRNA and protein levels was restored using pcDNA3.1/ITPR1 in U2OS cells with LINC00662 silencing (^*∗∗*^*P* < 0.01; Figure 5(a)). As depicted in Figures 5(b) and 5(c), overexpression of ITPR1 significantly restored the LINC00662 silencing-suppressed proliferative activity of U2OS cells (^*∗∗*^*P* < 0.01). Moreover, ITPR1 overexpression abrogated the suppressive effect of LINC00662 depletion on cell invasion and migration (^*∗∗*^*P* < 0.01; Figures 5(d) and 5(e)). Meanwhile, the inhibition of LINC00662 knockdown on stemness in U2OS cells was reversed by ITPR1 overexpression (^*∗∗*^*P* < 0.01; Figures 5(f) and 5(g)). Overall, ITPR1 is involved in the LIN00662-mediated regulation of OS cell functions.

## 4. Discussion

In recent years, increasing evidence has revealed that lncRNAs could exert important regulatory functions on the occurrence and development of various cancers, including OS [9–11, 13, 18], demonstrating the potential research value of lncRNAs in the molecular mechanisms underlying cancer progression. The biological roles of LINC00662 have been studied in a few types of cancers. For example, LINC00662 facilitates cell migration, invasion, and stemness maintenance via interacting with LIN28 in lung cancer [19]. Inhibition of LINC00662 reduces cell proliferative, migratory, and invasive abilities of in oral squamous cell carcinoma [20]. LINC00662 mediates the malignant phenotypes of colorectal cancer cells by the miR-145/c-myc axis [21]. In our research, we demonstrated that LINC00662 was significantly upregulated in OS tissues and cell lines and was negatively related to the overall survival of patients with OS. Then, we found that depletion of LINC00662 significantly inhibited OS cell proliferation, migration, invasion, and stem cell properties *in vitro*. These findings exhibited the carcinogenic role of LINC00662 in OS, which is consistent with the previous studies.

There has been increasing attention drawn to the theory of ceRNA network; that is, lncRNAs function as sponges for miRNAs to modulate the expression of the target genes [22]. Accumulating research has indicated that lncRNAs could exert ceRNA functions in cancers. For example, lncRNA LINC00460 enhances the progression of head and neck squamous cell carcinoma through sponging miR-612 and upregulating AKT2 [23]. LncRNA SPRY4-IT1 binds to miR-6882-3p by competing with TCF7L2 to affect the cell stemness of breast cancer [24]. LncRNA CHL1-AS1 increases the abilities of cell proliferation and migration via sponging miR-6076 to upregulate CHL1 expression in endometrial cancer [25]. In addition, LINC00662 has been reported to serve as a ceRNA in some cancers [26, 27]. In this study, we examined the subcellular localization of LINC00662 in OS cells and found that LINC00662 was mostly distributed in the cytoplasm, suggesting the possibility that LINC00662 might be involved in ceRNA network by sponging miRNAs. MicroRNAs, a group of small ncRNA molecules with 20–24 nucleotides, can prevent the translation or degradation of target mRNAs at the posttranscriptional level [28]. Through online tools, miR-16-5p was predicted to bear the complementary base pairing with LINC00662. In this study, LINC00662 was validated to bind to miR-16-5p. Previous investigations have shown that miR-16-5p is differentially expressed and plays a key role in the progression of numerous cancers, such as breast cancer [29], cervical cancer [30], and hepatocellular carcinoma [31]. In the present study, miR-16-5p was expressed at a low level in OS tissues and cells and negatively related to LINC00662 in terms of expression level. Conclusively, LINC00662 functions as a molecular sponge for miR-16-5p in OS cells.

To further support the ceRNA pattern in OS, the target genes of miR-16-5p were investigated. Based on the bioinformatics tools, ITPR1 (inositol 1,4,5-trisphosphate receptor, type 1) was identified. ITPR1 has been reported to be involved in Ai-lncRNA EGOT-mediated paclitaxel resistance in human cancers [32]. ITPR1 serves as a direct target of EPAS1 and an autophagy regulator to protect renal carcinoma cells against NK-mediated killing [33]. In this study, ITPR1 was verified to be a direct target of miR-16-5p. Meanwhile, ITPR1 was highly expressed in OS tissues and cells. In addition, rescue assays confirmed that overexpression of ITPR1 could reverse the LINC00662 silencing-induced inhibitive effect on OS cell functions.

In conclusion, this study investigated the biological role and regulatory mechanisms of LINC00662 in OS. Our data support evidence that LINC00662 exerts ceRNA function by sponging miR-16-5p to upregulate ITPR1, thereby mediating the malignant phenotypes of OS cells. Our findings might provide an instructive insight for the future exploration of OS treatment.

## Figures and Tables

**Table 1:** Correlation between the LINC00662 expression and the clinicopathological characteristics of osteosarcoma patients.

Characteristics	Cases	LINC00662 expression		*P* value
		High *n* = 26	Low *n* = 25	
*Gender*				
Male	30	17	13	0.332
Female	21	9	12	

*Age*				
≤60	25	12	13	0.676
>60	26	14	12	

*Morphological type*				
Classic central OS	11	7	4	0.829
Intraosseous well-differentiated OS	9	5	4	
Parosteal juxtacortical OS	13	6	7	
Periosteal OS	8	3	5	
High-grade surface OS	10	5	5	

*Anatomic location*				
Tibia/femur	39	21	18	0.342
Elsewhere	12	5	7	

*Distant metastasis*				
Yes	31	22	9	0.000
No	20	4	16	

*TNM stage*				
I + II	27	7	20	0.000
III + IV	24	19	5	

*Tumor size (cm)*				
≤5	19	5	14	0.007
>5	32	21	11	

*P* < 0.05 is considered statistically significant (Chi-squared test).

**Table 2:** Univariate and multivariate Cox analyses of various potential prognostic factors for overall survival in osteosarcoma patients.

Variables	Univariate analysis			Multivariate analysis		
	HR	95% CI	*P* value	HR	95% CI	*P* value
Gender	0.663	0.226–1.944	0.454			
Age	4.108	0.888–19.004	0.071			
Pathological type	0.681	0.200–2.320	0.539			
Anatomic location	1.261	0.379–4.192	0.705			
Distant metastasis	5.293	1.519–18.441	0.009	4.800	1.421–16.216	0.012
TNM stage	0.236	0.059–0.938	0.040	0.324	0.097–1.081	0.067
Tumor size (cm)	0.131	0.025–0.679	0.015	0.315	0.093–1.073	0.065
LINC00662 expression	0.199	0.050–0.796	0.023	0.212	0.062–0.723	0.013

HR: hazard ratio; CI: confidence interval. *P* < 0.05 is considered statistically significant (Chi-squared test).

## Data Availability

The datasets used or analyzed during the current study are available from the corresponding author on reasonable request.

## Supplementary Materials

Supplementary Materials Supplementary Figure 1: design of the present study.
