# Supplementary figures and images for: LncRNA LINC00662 Exerts an Oncogenic Effect on Osteosarcoma by the miR-16-5p/ITPR1 Axis
Source: J Oncol. 2021 Sep 28;2021:8493431. doi: 10.1155/2021/8493431 (PMC8492273; doi:10.1155/2021/8493431)

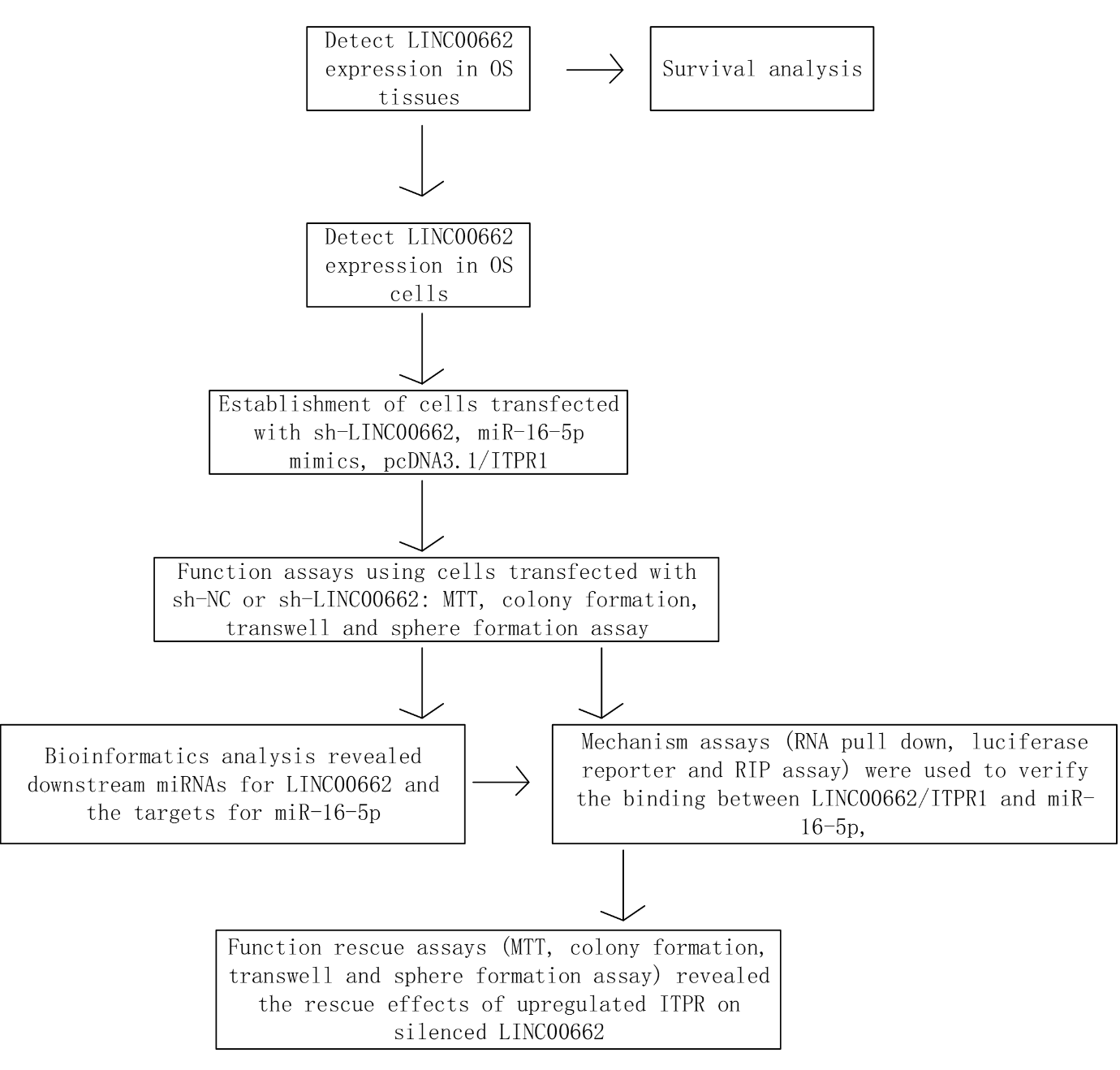


**Supplementary Figure 1 Design of the present study.**

Supplement: Supplementary Materials — Supplementary Figure 1: design of the present study. [file 8493431.f1.docx]
